# The Effect of Hemodynamic Parameters on Cerebral Oxygenization During Carotid Endarterectomy

**DOI:** 10.21470/1678-9741-2020-0398

**Published:** 2022

**Authors:** Cihan Yücel, Serkan Ketenciler, Mete Gürsoy, Seray Türkmen, Nihan Kayalar

**Affiliations:** 1 Department of Cardiovascular Surgery, Okmeydanı Training and Research Hospital, Istanbul, Turkey.; 2 Department of Anesthesia and Intensive Care, Okmeydanı Training and Research Hospital, Istanbul, Turkey.

**Keywords:** Atherosclerosis. Carotid Endarterectomy. Near-Infrared Spectroscopy. Carotid Artery, Common. Anesthesia, General

## Abstract

**Objective/Introduction:**

Near-infrared spectroscopy (NIRS) is a non-invasive technique to detect cerebral ischemia by monitoring changes in regional cerebral oxygenation (rSO_2_) in the frontal lobes. However, there are no studies showing the changes in NIRS values in response to hemodynamic variations during stages of carotid endarterectomy (CEA) procedure and clinical implications of these changes. The aim of this study was to determine if hemodynamic changes affect NIRS values during carotid endarterectomy and if our results may help to provide strategies for hemodynamic management in these patients.

**Methods:**

A total of 50 consecutive patients undergoing CEA were prospectively included in the study. NIRS was measured at first minute after clamping of carotid artery, and then systolic blood pressure was increased above 150 mmHg. NIRS values from both hemispheres were recorded simultaneously at certain time points and were analyzed to evaluate the changes at different stages of operation and to assess correlations with hemodynamic parameters.

**Results:**

NIRS values on the right and left sides were correlated with systolic (right *P*<0.001, R2:0.24; left *P*=0.02, R2:0.10) diastolic (right *P*<0.001, R2:0.36; left *P*=0.001, R2:0.18) and mean (right *P*<0.001, R2:0.33; left *P*=0.003, R2:0.17) blood pressures when the patient was under general anaesthesia. NIRS values were significantly lower than pre-incision values just after clamping of carotid artery in both hemispheres (*P*=0.005 for the right and *P*<0.001 for the left side).

**Conclusion:**

NIRS values measured in our study show that there is a correlation between hemodynamic changes and cerebral oxygenation. This effect is especially pronounced while the patient is asleep and intubated, which implies the importance of close monitoring of patients with carotid disease during any surgery requiring general anaesthesia.

**Table:** 

Abbreviations, acronyms & symbols	
CC	= Cross-clamping
CEA	= Carotid endarterectomy
ECG	= Electrocardiography
EEG	= Electroencephalography
MEP	= Motor-evoked potential
NIRS	= Near-infrared spectroscopy
rSO_2_	= Regional cerebral oxygenation
SPSS	= Statistical Package for the Social Sciences
SSEP	= Somatosensory-evoked potentials

## INTRODUCTION

Carotid endarterectomy (CEA) is a well-established procedure to prevent development of future stroke in symptomatic and asymptomatic patients with a high-grade internal carotid artery stenosis ^[1,2]^. On the other hand, the procedure itself carries a risk of stroke, which can be due to thrombosis, embolism, or intraoperative ischemia related to hypoperfusion during cross-clamping (CC) of the carotid artery ^[3]^. Therefore, monitoring techniques to determine adequacy of cerebral circulation are used and include electroencephalography (EEG), somatosensory-evoked potentials (SSEP), motor-evoked potential (MEP), transcranial Doppler, stump pressure and cerebral oximetry ^[4]^. 

Near-infrared spectroscopy (NIRS) is a non-invasive technique to detect cerebral ischemia by monitoring changes in regional cerebral oxygenation (rSO_2_) in the frontal lobes. NIRS is easy to apply and has a relatively low cost compared to other non-invasive techniques. Various studies compared NIRS with stump pressure ^[5,6]^, EEG ^[7]^, and transcranial Doppler ^[8]^ and proposed it as a reliable and safe substitute for these traditional techniques. However, there are no studies showing the changes of NIRS values in response to hemodynamic variations during stages of CEA procedure and clinical implications of these changes. Some surgeons believe that an increase in mean arterial blood pressure, usually by 20% or systolic arterial blood pressure above 150 mmHg during CC of carotid artery is quite enough to prevent cerebral ischemia. Nevertheless, there is no scientific data supporting this practice. In the current study, we evaluated the NIRS values at certain stages during carotid endarterectomy and their relation to hemodynamic changes at these time points. The aim was to determine if hemodynamic changes affect NIRS values while the other factors, such as arterial oxygen saturation, were kept relatively constant. We also intended to provide a scientific basis for the increased blood pressure protocol during carotid endarterectomy, hoping that our results will be helpful for future hemodynamic management protocols for patients with carotid stenosis.

## METHODS

Between October 2018 and September 2019, 50 consecutive patients undergoing CEA under general anaesthesia were prospectively included in the study. The hospital ethics committee approved the study (EC number: 2018-973), and the individual informed consent was waived. Indications for CEA were symptomatic or asymptomatic carotid stenosis >70% or symptomatic patients with 50-69% carotid stenosis, as stated in guidelines ^[1,2]^ and discussed by the team. Carotid stenting was preferred over endarterectomy when revascularization is indicated in patients with cervical anatomy unfavorable to arterial surgery and at high surgical risk, but these patients were not included in the study. Patients who underwent elective carotid surgery were included and those with recent cerebral events were excluded from the sudy. Patients with either unilateral or bilateral lesions were included in the study but those with chronic total occlusion of the carotid artery were not operated on. In patients with bilateral lesions, the side of CEA was determined by the dominant hemisphere or the severity of the lesion as well as the side of symptoms, if any. The severity of the carotid artery stenosis was initially assessed by carotid Doppler ultrasound imaging and confirmed by computed tomography angiography in all patients. We included patients who underwent CEA with concomitant coronary surgery because our instituitional approach is to perform the carotid part of the operation before the coronary grafting and, therefore, measurements are logically not affected by parameters related to coronary surgery. 

### Operative and Postoperative Protocol

All operations were done by the same team. CEA was performed under general anesthesia in all patients in accordance to the surgeon’s preference. Patients at high risk for general anaesthesia and others who underwent operation with regional anaesthesia were not included in the study to provide a more homogeneous group. Routine monitoring with ECG and pulse oximetry were used. Intra-arterial blood pressure was measured and recorded continuously using a 20-gauge catheter placed in the radial artery. Heparin (5000 U) was administered intravenously 3 minutes before CC of the carotid artery. Activated clotting time was used to monitor heparin treatment with the aim of a value above 200 seconds and an additional dose was administered when necessary. A standard longitudinal endarterectomy was performed in all patients with primary or patch closure of arteriotomy, depending on the arterial diameter and the surgeon’s preference. Our strategy to use shunting is when NIRS values drop 20-25% from baseline or when the clamp time is anticipated to be long due to the anatomy of the patient and the carotid lesion. Clamp time is the duration from the start of internal carotid artery clamping to the removal of this clamp and operation time is the duration from the start of carotid surgery skin incision to the closure of this incision. In patients with concomitant coronary artery bypass grafting, CEA procedure was completed before cannulation for cardiopulmonary bypass. 

Initially, patients’ blood pressure was regulated and kept within normal limits and hypertension was avoided. After CC of the carotid artery, NIRS at first minute was measured and then systolic blood pressure was increased above 150 mmHg and vasoactive medications were used, if required. Ephedrine was used as the preferred vasoactive medication ^[9]^. After CC was removed, blood pressure was regulated to keep it within normal limits. Patients were extubated after the return of spontaneous respiration and were transferred to the intensive care unit for follow-up on the first night. All patients were on dual antiplatelet therapy, with 75 mg of acetylsalicylic acid and clopidogrel, in the postoperative period.

### NIRS Measurements

Continuous bilateral rSO_2_ measurements were performed using a Masimo Cerebral Oximeter (Masimo Corp, CA, USA), by 2 sensors placed on the forehead. NIRS values from both hemispheres were recorded simultaneously at certain time points and were compared with each other to evaluate the changes at different stages of operation as follows: before the start of the operation, when the patient was awake, after the patient is asleep just before incision, at the first minute of CC of carotid artery, just after increasing blood pressure, every 5 minutes after CC, after removal of CC and when the patient is awake and extubated. All these values were compared ipsilaterally with each other (right NIRS *vs*. right NIRS) and also right and left NIRS values were compared with each other at each time point. 

### Statistical Analysis

Statistical analysis was performed with IBM SPSS software version 20 (IBM, Armonk, NY, USA). All data are presented as mean ± standard deviation for continuous variables, as numbers with percentages for categorical variables. The Shapiro-Wilk test was used to evaluate the normality of variable distribution. In the comparison of two independent groups, the Student’s t-test was used for numerical variables with normal distribution. The correlations of systolic, diastolic and mean pressures with NIRS at different time points were investigated using Pearson’s correlation coefficient and Bland-Altman analysis. A *P* value of 0.05 or less was considered significant.

## RESULTS

### Patient Characteristics

A total of 50 consecutive patients who underwent CEA under general anaesthesia between October 2018 and September 2019 were prospectively included in the study. Data for all patients were collected prospectively and were complete. The mean age was 69.04±6.6 (range, 54-82 years) and most patients had a risk factor for atherosclerosis (Table 1). Most patients had CEA for symptomatic carotid disease (stroke, 38% or transient ischemic attack, 20%), as shown in Table 1. Concomitant coronary artery bypass grafting was performed in 6 (12%) patients. 

**Table 1:** Preoperative patient data.

Age (years)		69.04±6.6	54-82
Ejection fraction		55.4±5.7	45-65
**Variable**		**n**	** % of total**
Sex	Male	34	32
	Female	16	
Hypertension		37	74
Smoking		33	66
Diabetes mellitus		23	46
Coronary artery disease		10	20
Concomitant CABG		6	12
COPD		11	22
Symptom	Stroke	19	38
	TIA	10	20
	Asymptomatic	21	42
Bilateral lesion		18	36

CABG=coronary artery bypass grafting; COPD=chronic obstructive pulmonary disease; TIA=transient ischemic attack

### Perioperative Data

All patients underwent either right (44%) or left (56%) CEA under general anaesthesia. In 18 patients with bilateral lesions, a left CEA was performed in most patients (14). Primary closure of the arteriotomy was preferred in most patients (78%) and shunt was not used in any patient. Postoperatively, there was transient hemiparesis in 1 patient and local hematoma of the surgical site was observed in 4 patients, 2 of whom required re-exploration. The patient with transient hemiparesis underwent left CEA and woke from anaesthesia with right-sided hemiparesis that lasted 2 days. At the time of operation of this patient, NIRS values showed a decline of about 25% from baseline 5 minutes after clamping, but it was not possible to insert a shunt due to anatomical problems. The CT scan of this patient showed no pathology and the patient was discharged without any neurological deficits. There were no major stroke, nerve injury or death in any other patient. Surgical and early postoperative data are summarized in Table 2. 

**Table 2:** Operative and postoperative patient data.

	Variable	n	% of total
Side of endarterectomy	Right	22	44
	Left	28	56
Arteriotomy closure	Primary	39	78
	Patch	11	22
Shunt use		0	0
Postoperative complications	Permanent cerebrovascular event	0	0
	Transient hemiparesis	1	2
	Local hematoma	4	8
	Exploration for bleeding/hematoma	2	4
	Mortality	0	0
**Variable**		**Mean±SD (range)**	
Clamp duration (minutes)		21.7±6.3 (9-33)	
ICU stay (days)		1.1±0.5 (1-3)	
Hospital stay (days)		3.3±1.3 (2-8)	

ICU=intensive care unit

### NIRS Assessments

NIRS values of the right and left hemispheres were compared with each other and were not different from each other at any of the time points (Table 3). We also analyzed these differences in patients with either right, left or bilateral lesions and could not find any difference at any time point, so we did not present them in the table to simplify the interpretation of results. 

**Table 3:** Comparison of NIRS values at different time points.

Time NIRS measured	Right-sided NIRS	Left-sided NIRS	*P*-value
Before intubation	60.5±5.3	59.7±3.7	0.2
Before incision	59.3±7.5	60.4±7.5	0.1
*P*-value	0.2	0.4	
Before incision	59.3±7.5	60.4±7.5	0.1
1 minute after clamping	56.7±8.3	56.5±8.4	0.8
*P*-value	**0.005**	** <0.001 **	
1 minute after clamping	56.7±8.3	56.5±8.4	0.8
After SBP above 150 mmHg	58.5±7.9	57.7±10.1	0.4
*P*-value	**0.001**	0.2	
Before intubation	60.5±5.3	59.7±3.7	0.2
After SBP above 150 mmHg	58.5±7.9	57.7±10.1	0.4
*P*-value	0.054	0.1	
After SBP above 150 mmHg	58.5±7.9	57.7±10.1	0.4
5 minutes after clamping	56.6±7.8	55.4±10.1	0.2
*P*-value	0.8	** <0.001**	
5 minutes after clamping	56.6±7.8	55.4±10.1	0.2
15 minutes after clamping	58.3±7.6	59.1±8.8	0.4
*P*-value	** <0.001**	**<0.001**	
Before incision	59.3±7.5	60.4±7.5	0.1
After removing clamp	59.8±7.4	60.2±8.3	0.5
*P*-value	0.5	0.7	
Before intubation	60.5±5.3	59.7±3.7	0.2
After extubation	62.3±7.6	62.6±7.2	0.6
*P*-value	0.07	0.002	

Before incision=patient is asleep and intubated; Before intubation=patient is awake; NIRS=near-infrared spectroscopy; SBP=systolic blood pressure

NIRS values were observed to drop significantly from pre-incision values just after CC of carotid artery in both hemispheres (*P*=0.005 for the right side and *P*<0.001 for the left side). At this point, blood pressures were being regulated to keep them within normotensive limits. Systolic blood pressures increased above 150 mmHg and NIRS values were observed to increase immediately and were comparable to values before intubation and incision, as seen in Table 3. At the 5^th^ minute after CC, NIRS values were observed to fall from the values in systolic blood pressures of 150 mmHg (*P*=0.8 for the right side and *P*<0.001 for the left side). However, they increased and were stable after 10 minutes of CC of carotid artery. In Table 3, 15-minute values were given as representative of these similar and stable values. 

Although NIRS values decreased significantly after CC, in only six patients they dropped to more than 20% of baseline values. Three of these patients underwent right CEA, 3 underwent left CEA and only 1 had bilateral lesions. The decrease in NIRS values was observed ipsilateral to CEA in all. In 5 of these patients, after the increase in systolic blood pressure levels, NIRS values increase to levels above 80% of the initial measurements. In 1 patient, NIRS level remained low, but due to technical reasons, it was not possible to insert a shunt and this was our only patient with postoperative transient hemiparesis. 

After completing the procedure and removing CC of carotid arteries, NIRS values increased to pre-incision levels. When compared to preoperative awake values, postoperative awake values did not increase significantly on the right side, but were found to be increased on the left (*P*=0.02). 

**Fig. 1:**
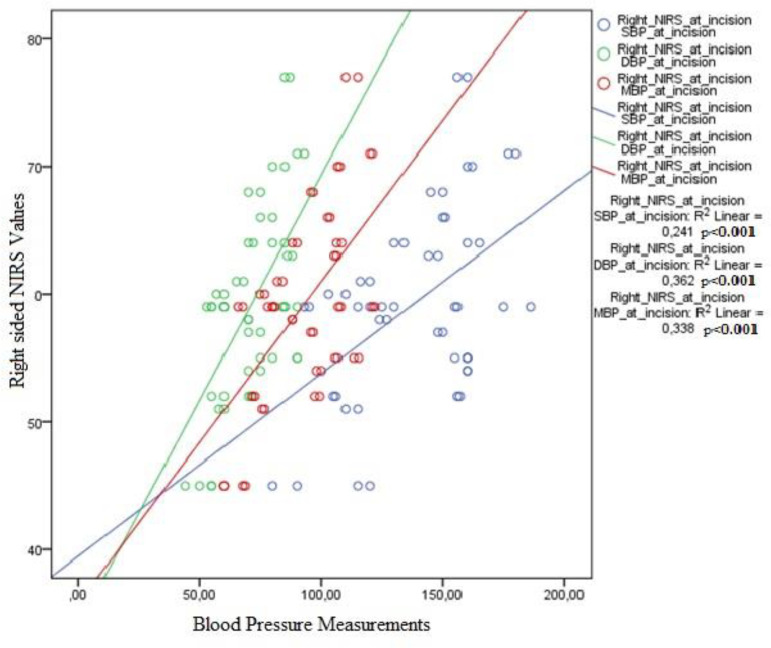
Correlation of right-sided NIRS values with blood pressure indices after general anaesthesia.

**Fig. 2:**
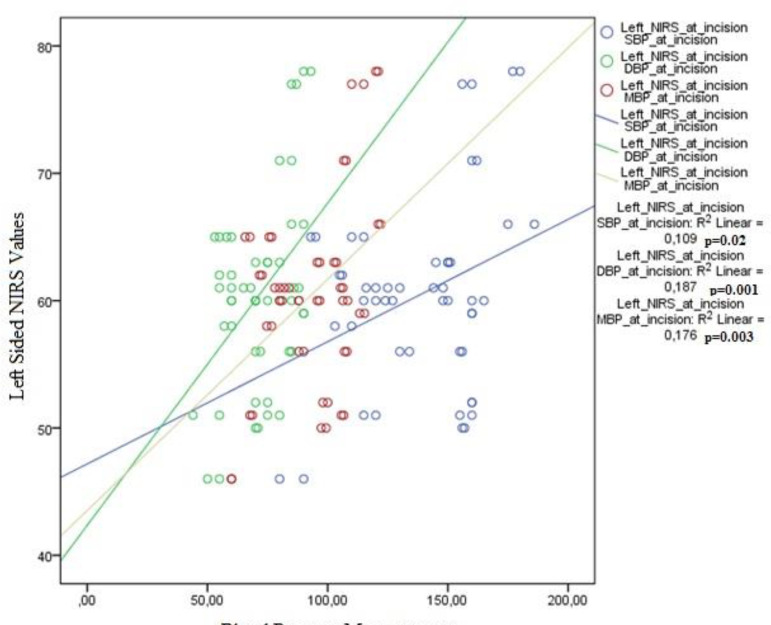
Correlation of left-sided NIRS values with blood pressure indices after general anaesthesia.

Correlation of NIRS values with blood pressure indices at different time points was evaluated to assess if hemodynamic changes are more effective at any stage of the operation. Right- and left-sided NIRS values were well correlated with systolic, diastolic and mean blood pressures when the patient is asleep and intubated (Figures 1 and 2). When patients were awake or when carotid artery was clamped during surgery, the correlations were not statistically significant. 

## DISCUSSION

Cerebral oximetry measures the ratio of oxygenated hemoglobin to total hemoglobin and the results can be influenced by many factors, including head position, mean arterial pressure, systemic arterial blood oxygen saturation and partial pressure of carbon dioxide, hemoglobin concentration, brain tissue oxygen consumption, and intracranial pressure ^[10]^. Nevertheless, it has been used as a reliable and safe substitute for traditional brain monitoring techniques. Wang et al. ^[11]^ found that the sensitivity and specificity of NIRS monitoring for intraoperative hypoperfusion were 64.3% and 90.0%, resulting in a strong consistency with transcranial Doppler ultrasonography monitoring results. Jonsson et al. ^[5]^ compared stump pressures and NIRS measurements in CEA operations in terms of shunt placement and reported that NIRS monitoring has a high sensitivity and acceptable specificity in predicting cerebral ischaemia and can be an attractive alternative for stump pressure measurement. One of the major advantages of NIRS monitoring is the spontaneous observation of the changes in cerebral oxygenation in accordance to changes in cerebral circulation. These properties, along with its ease of use, rendered NIRS monitoring a very suitable tool for this study, because it was possible to assess spontaneous changes in cerebral oxygenation in response to hemodynamic changes. 

In our institution, carotid operations are performed under either general or local anaesthesia with sedation. There are many factors affecting NIRS values as mentioned above and most of these, such as oxygen saturation and partial pressure of carbon dioxide in systemic arterial blood, as well as blood pressure can be easily controlled under general anaesthesia. Therefore, patients undergoing CEA under general anaesthesia created a homogenous group to analyze mostly the effects of hemodynamic changes on NIRS and were specifically selected for this study. Further studies including patients undergoing surgery with local anaesthesia may be performed and may give further insight about the cerebral oxygenation during various stages of carotid surgery in different patient groups. 

In this study, NIRS values were only found to be correlated with blood pressure indices when the patient was under general anaesthesia (Figures 1 and 2). This is an important finding that shows that regulatory mechanisms of cerebral blood flow are less effective and cerebral oxygenation is more dependent on hemodynamic changes in patients under general anaesthesia. It is not possible to compare this result with any other, since there are no studies in the literature that evaluate the effects of hemodynamic parameters on cerebral oxygenation by any methods of cerebral monitoring. This study is unique in this regard and may lead to future studies that use and compare different methods of cerebral monitoring to assess factors affecting cerebral oxygenation in patients with carotid disease undergoing any surgery under general anaesthesia. 

A time-honored approach to maintain cerebral perfusion during CC of the carotid artery is to increase mean arterial blood pressure, usually by 20% or systolic blood pressure above 150 mmHg. Although this is a general discussion point among surgeons, there are no studies in the literature that quantitatively assess the effect of increased blood pressure on cerebral perfusion during carotid artery clamping. This study showed that initial decrease in NIRS values after CC was restored by increasing systolic blood pressure above 150 mmHg (Table 3). This suggests that blood pressure has an influence on brain oxygenation whereas NIRS values did not correlate with blood pressure indices after CC of the carotid artery in our analysis. This may be because systolic blood pressures were kept stable and around 150-160 mmHg in all our patients from this time until the removal of carotid clamp. Since there are no comparable studies, this may be evaluated in another study without strict control of blood pressure after CC of the carotid artery.

There was a second decrease in the NIRS values on the left side 5 minutes after clamping, which increased in a very short period and remained so till the clamp was removed. These values are all analyzed on a minute basis and the representative values are presented in Table 3. They remain stable afterwards at stable blood pressures, which suggests that local regulation of cerebral blood flow may also have a role. The reason for observing this only on the left side is difficult to interpret, but it may be the result of more patients undergoing CEA on the left carotid artery.

Although this study showed a positive relationship between hemodynamic parameters and NIRS values, the clinical implications of these findings are still controversary. Percent changes in NIRS values after CC of carotid artery and increased blood pressures were relatively small in our study. The cut-off value of NIRS varies among different clinical settings. Although a 12% decrease in rSO_2_ was reported to have a higher sensitivity, specificity, and predictive values than a 20% decrease in rSO_2_^[12]^, it was recently agreed that more than a 20% decrease in rSO_2_ compared to the baseline value indicates a need for intervention ^[13,14]^ and could be considered as a cut-off value to indicate cerebral ischemia ^[15]^. We observed a decrease in NIRS values of more than 20% in 6 patients and in 5 of them this percentage decreased to less than 20% after increasing blood pressure and in the remaining patient we observed a neurological complication. Although our numbers are low to achieve a statistical significance, we can suggest that decreased NIRS values may indicate a neurological complication if they remain below 20% of baseline values after the increase in blood pressure.

Another finding in the present study was the correlation of the right- and left-sided NIRS values with each other, regardless of the side of the lesion. This was true for all time points of measurement, including those after clamping. Moreover, 18 patients with bilateral lesions showed similar pattern of NIRS changes to the patients with unilateral lesions (*e.g*. right- and left-sided NIRS in those with bilateral lesions; at incision, 60±7.5 and 62.3±7.1; after CC of the carotid artery, 56.1±7.6 and 56.2±7.6). Only in those patients with more than 20% decrease in NIRS values, the decrease was ipsilateral to the clamped carotid artery in all. It is possible that this decrease occurs in patients with problems of intracerebral circulation and in patients with an intact circle of Willis, the cerebral oxygenation is affected bilaterally or globally after clamping one side, regardless of the side. An anatomical study with a larger number of patients that includes screening the completeness of circle of Willis is necessary to prove this finding. 

One important shortcoming of this study is the relatively small number of patients. Since the number of postoperative neurological complications is very low, it is also not possible to interpret the full clinical implication of our findings. Moreover, there are no similar studies to compare our results, which on the other hand makes the study unique. Authors are also aware that NIRS monitoring has its shortcomings and the results may be affected by many factors, as stated. However, this is a preliminary study with important results and we believe it will lead to further studies to assess the factors affecting cerebral oxygenation during carotid endarterectomy and their clinical implications.

## CONCLUSION

As an acceptable monitoring tool during CEA, NIRS values show a correlation between hemodynamic changes and cerebral oxygenation. This effect is especially pronounced while the patient is asleep and intubated, which implicates the importance of close monitoring of patients with cartotid disease during any surgery that requires general anaesthesia.

**Table:** 

Authors' roles & responsibilities	
CY	Substantial contributions to the conception or design of the work; or the acquisition, analysis, or interpretation of data for the work; drafting the work or revising it critically for important intellectual content; agreement to be accountable for all aspects of the work in ensuring that questions related to the accuracy or integrity of any part of the work are appropriately investigated and resolved; final approval of the version to be published
SK	Substantial contributions to the conception or design of the work; or the acquisition, analysis, or interpretation of data for the work; drafting the work or revising it critically for important intellectual content; agreement to be accountable for all aspects of the work in ensuring that questions related to the accuracy or integrity of any part of the work are appropriately investigated and resolved; final approval of the version to be published
MG	Substantial contributions to the conception or design of the work; or the acquisition, analysis, or interpretation of data for the work; drafting the work or revising it critically for important intellectual content; agreement to be accountable for all aspects of the work in ensuring that questions related to the accuracy or integrity of any part of the work are appropriately investigated and resolved; final approval of the version to be published
ST	Substantial contributions to the conception or design of the work; or the acquisition, analysis, or interpretation of data for the work; drafting the work or revising it critically for important intellectual content; agreement to be accountable for all aspects of the work in ensuring that questions related to the accuracy or integrity of any part of the work are appropriately investigated and resolved; final approval of the version to be published
NK	Substantial contributions to the conception or design of the work; or the acquisition, analysis, or interpretation of data for the work; drafting the work or revising it critically for important intellectual content; agreement to be accountable for all aspects of the work in ensuring that questions related to the accuracy or integrity of any part of the work are appropriately investigated and resolved; final approval of the version to be published
